# Accidental Administration of Anti-D Immunoglobulin to an Rh(D)-Positive Neonate: A Case Report

**DOI:** 10.7759/cureus.102746

**Published:** 2026-01-31

**Authors:** Motohisa Oteki, Mari Hayata, Tomonori Ichikawa

**Affiliations:** 1 Department of Neonatology, Kawaguchi Municipal Medical Center, Kawaguchi, JPN; 2 Department of Neonatology, Institute of Science Tokyo, Bunkyo, JPN

**Keywords:** anti-d immunoglobulin, direct coombs test, hemolytic anemia, hyperbilirubinemia, neonate

## Abstract

Anti-D immunoglobulin (RhIg) is administered to Rh(D)-negative mothers to prevent alloimmunization in Rh(D)-incompatible pregnancies, and is contraindicated in Rh(D)-positive individuals. Herein, we report a case of accidental intramuscular administration of RhIg to an Rh(D)-positive neonate.

A female infant was born at 39 weeks of gestation with a birth weight of 2,432 g. Shortly after birth, one vial of RhIg (250 μg), which should have been administered to the mother, was mistakenly injected into the neonate. The infant was transferred to our neonatal intensive care unit at one day of age for careful observation. On admission, the infant was in good general condition without pallor or jaundice. Laboratory tests revealed a hemoglobin level of 16.9 g/dL and a total bilirubin level of 4.54 mg/dL. The direct Coombs test was positive (2+). During hospitalization and subsequent outpatient follow-up, hemoglobin and bilirubin levels remained within the reference ranges, and neither phototherapy nor transfusion was required. The direct Coombs test remained positive for approximately three months, after which it spontaneously became negative.

Although theoretical concerns exist regarding hemolysis after RhIg administration in Rh(D)-positive neonates, severe hemolytic complications were not observed in this case. Based on our case and previous reports, careful short-term observation followed by outpatient follow-up appears to be a reasonable management strategy for similar accidental administrations.

## Introduction

Anti-D immunoglobulin (RhIg) is a plasma-derived blood product indicated for Rh(D)-negative pregnant women to prevent maternal alloimmunization in Rh(D)-incompatible pregnancies. Administration of RhIg at approximately 28 weeks of gestation and postpartum suppresses anti-D antibody production, thereby preventing hemolytic disease in the fetus and newborn in subsequent pregnancies [1].

Conversely, RhIg is contraindicated in Rh(D)-positive individuals because of the theoretical risk of hemolysis and hemolytic jaundice. Reports on inadvertent RhIg administration in Rh(D)-positive neonates are limited. To our knowledge, no cases with a severe clinical course have been reported, and the most significant intervention described has been phototherapy for moderate hyperbilirubinemia [2-10].

Herein, we present a case in which RhIg intended for the mother was inadvertently administered intramuscularly to an Rh(D)-positive neonate. Although the direct antiglobulin test (DAT) remained positive for an extended period, the infant did not develop overt hemolytic anemia or significant hyperbilirubinemia. The clinical course is described and compared with that of previously reported cases.

## Case presentation

A one-day-old female neonate was referred to our neonatal intensive care unit after accidental administration of RhIg. The mother was 36 years old, gravida three, para zero, with blood type A and Rh(D)-negative status, and irregular antibody screening was negative. The pregnancy course was unremarkable, and there was no evidence of maternal infection. Family history was notable for bipolar disorder in the father and Rh(D)-negative blood type in the mother, and no hematological disorders were identified.

The infant was delivered vaginally at 39 weeks and two days of gestation, with Apgar scores of 9 and 10 at one and five minutes, respectively. Anthropometric measurements at birth were as follows: weight 2,432 g (−1.48 SD), length 47.5 cm (−0.81 SD), and head circumference 33.0 cm (−0.19 SD). On the day of birth, a single vial of RhIg (250 μg anti-D), which had been intended for the mother, was inadvertently administered intramuscularly to the neonate. The infant was subsequently transferred to our neonatal intensive care unit for monitoring at approximately 23 hours after the injection.

Upon admission, vital signs were stable: body temperature 37.6°C, heart rate 145 beats per minute, respiratory rate 30 breaths per minute, blood pressure 58/24 mmHg, and oxygen saturation 95% on room air. The infant appeared well and active, without pallor or jaundice, and physical examination revealed no abnormalities in the cardiovascular or respiratory systems.

Laboratory findings obtained approximately 24 hours after RhIg administration are summarized in Table 1. The hemoglobin level was 16.9 g/dL, the total bilirubin level was 4.54 mg/dL, and the carboxyhemoglobin level was 1.0%. Blood typing confirmed an AB and Rh(D)-positive status. The DAT was positive (2+); however, there were no clinical or laboratory findings suggestive of hemolysis.

**Table 1:** Laboratory data on admission WBC: white blood cell count, RBC: red blood cell count, Hb: hemoglobin, Hct: hematocrit, Plt: platelet count, Retic: reticulocytes, TP: total protein, Alb: albumin, LDH: lactate dehydrogenase, AST: aspartate aminotransferase, ALT: alanine aminotransferase, T-bil: total bilirubin, D-bil: direct bilirubin, BUN: blood urea nitrogen, Cr: creatinine, Na: sodium, K: potassium, Cl: chloride, Ca: calcium, CRP: C-reactive protein, pH: potential of hydrogen, pCO₂: partial pressure of carbon dioxide, HCO₃⁻: bicarbonate, BE: base excess, Lac: lactate, COHb: carboxyhemoglobin, DAT: direct antiglobulin test

Parameter	Value	Unit
WBC	21.0	×10^9/L
RBC	4.94	×10^12/L
Hb	16.2	g/dL
Hct	49.7	%
Plt	323	×10^9/L
Retic	3.9	%
TP	5.4	g/dL
Alb	3.4	g/dL
LDH	410	U/L
AST	39	U/L
ALT	7	U/L
T-bil	4.54	mg/dL
D-Bil	0.15	mg/dL
BUN	4.2	mg/dL
Cr	0.71	mg/dL
Na	145	mEq/L
K	4.3	mEq/L
Cl	110	mEq/L
Ca	9.4	mg/dL
CRP	0.02	mg/dL
pH	7.386	–
pCO2	44.0	mmHg
HCO3-	26.4	mmol/L
BE	0.8	mmol/L
Lac	2.7	mmol/L
COHb	1.0	%
ABO blood group	AB	–
Rh(D) status	Positive	–
DAT	2+	–

Hemoglobin and bilirubin levels were interpreted using standard age-adjusted neonatal reference ranges and were considered within physiological limits. Haptoglobin was not measured because it is physiologically low in neonates and is not considered a reliable marker of hemolysis in this age group. Peripheral blood smear examination did not reveal any abnormal red blood cell (RBC) morphology suggestive of hemolysis. Serum lactate dehydrogenase levels and reticulocyte counts were followed serially, and neither showed any increase suggestive of ongoing hemolysis.

**Figure 1:**
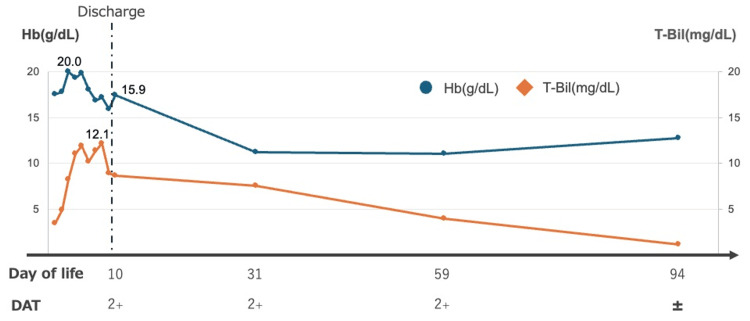
Time course of hemoglobin and total bilirubin with DAT status Hb: hemoglobin, T-bil: total bilirubin, DAT: direct antiglobulin test

During hospitalization and follow-up, hemoglobin and total bilirubin levels remained within physiological limits, and neither phototherapy nor blood transfusion was required. The DAT remained positive (2+) until approximately day 60 of life, decreased to trace positive (±) by day 96, and subsequently became negative without any intervention. No progressive anemia or prolonged jaundice was observed, and the overall clinical course was stable (Figure 1).

## Discussion

Summary of previously reported cases and clinical course

Table 2 summarizes the clinical courses of previously reported cases and our patient [2-10]. To our knowledge, no published reports of inadvertent RhIg administration in Rh(D)-positive neonates have documented severe hemolytic disease, and only two cases required phototherapy for moderate hyperbilirubinemia. Clinical outcomes have been consistently favorable across these cases, suggesting that the sequelae of accidental RhIg administration are relatively mild, irrespective of the dose administered or the infant's birth weight.

**Table 2:** Previously reported cases of inadvertent RhIg administration and the present case RhIg: anti-D immunoglobulin, Hb: hemoglobin, T-bil: total bilirubin, DAT: direct antiglobulin test, IM: intramuscular injection, SC: subcutaneous injection

Auther	Birth weight (g)	RhIg dose (µg)	Route	Clinical course/treatment	DAT
Niederhoff et al. (1969) [2]	3350	200	IM	No progression of anemia; T-bil increased to 15.0 mg/dL on day of life 2	Not reported
Marsh et al. (1970) [3]	2600	200	IM	No adverse events requiring treatment were reported	Not reported
Sanson and Veneziano et al. (1970) [4]	2700	300	IM	Moderate jaundice was reported	Positive
	Not reported	300	IM	No adverse events requiring treatment were reported	Positive
Chown et al. (1970) [5]	1500	150	IM	Hb decreased to 15.3 g/dL, and T-bil increased to 7.8 mg/dL within 1 week after birth	Positive
	3400	340	IM	No progression of anemia	Positive
Tanaka et al. (2001) [6]	2720	250	IM	No adverse events requiring treatment were reported	Positive/turned negative on day of life 109
Ukae et al. (2005) [7]	3000	62.5	IM	No progression of anemia; T-bil increased to 7.6 mg/dL on day of life 3	Not reported
Prasad (2006) [8]	Not reported	300	IM	Hb decreased to 15 g/dL, and T-bil increased to 18 mg/dL; phototherapy was administered on days 5–6 of life	Negative
Motonaga et al. (2020) [9]	3890	250	IM	No progression of anemia; T-bil increased to 11.4 mg/dL on day of life 3; phototherapy was given until day of life 4	Positive
	2590	250	SC	No progression of anemia; T-bil increased to 14.2 mg/dL on day of life 6	Positive
Pal et al. (2022) [10]	Not reported	300	IM	No progression of anemia; T-bil increased to 9.5 mg/dL on day of life 3	Negative
Present case (2025)	2432	250	IM	Hb decreased to 15.9 g/dL on day of life 9; T-bil increased to 12.1 mg/dL on day of life 8	2+/decreased to trace by day of life 92

Theoretically, the administration of RhIg to Rh(D)-positive neonates poses a risk of hemolysis, raising concerns regarding jaundice and progressive anemia development. Neither the previously reported cases nor our case demonstrated serious adverse events. This report discusses the factors that mitigate severe complications in Rh(D)-positive neonates and proposes an appropriate duration for post-exposure monitoring.

Quantitative considerations regarding hemolytic anemia

First, the theoretical extent of hemolysis relative to the RhIg dose was estimated. A 300 µg (1500 IU) dose of RhIg is sufficient to suppress alloimmunization after exposure to up to 15 mL of Rh(D)-positive RBCs, which is equivalent to 30 mL of whole blood [11].

Based on this estimate, a single vial of RhIg (250 µg) administered in our case could theoretically destroy approximately 12.5 mL of RBCs (25 mL of whole blood). This volume corresponds to approximately 8-10% of the total blood volume (approximately 240 mL) of a 3 kg neonate.

Assuming the complete hemolysis of this 8-10% fraction, the anticipated decrease in hemoglobin would be limited to approximately 1-2 g/dL. Given the physiological polycythemia characteristics of neonates, the clinical effect of such a decline is likely to be minimal. Furthermore, given the shorter lifespan of neonatal RBCs, the contribution of hemolysis to clinically significant anemia may have been less pronounced.

Hemolytic jaundice and immunological mechanisms

Regarding the risk of hemolytic jaundice, the catabolism of 1 g of hemoglobin yields approximately 36.2 mg of bilirubin [12]. Assuming a mean corpuscular hemoglobin concentration of 35 g/dL, the hemolysis of 1 mL of RBCs corresponds to 0.35 g of hemoglobin and would generate 12-13 mg of bilirubin. If the theoretical maximum volume of RBCs susceptible to one vial of RhIg (12.5 mL) were hemolyzed, the total bilirubin production would reach 150-160 mg. Given the immaturity of neonatal hepatic bilirubin metabolism, rapid destruction of this magnitude could theoretically precipitate significant hyperbilirubinemia.

However, our patient did not develop severe jaundice. A plausible explanation is the immaturity of the neonatal immune system and complement activity. Even when antibodies bind to the RBC surface, complement-mediated hemolysis may not progress readily, resulting in extremely slow RBC destruction [13]. Additionally, the IgG subclass distribution in RhIg resembles that of standard blood products; notably, IgG3, a subclass with relatively high hemolytic activity, accounts for only a small percentage of total IgG, which may further reduce the likelihood of severe hemolysis.

Duration of DAT positivity and RBC lifespan

In our case, the DAT remained positive for approximately three months. Tanaka et al. reported a case in which the DAT was negative 106 days after inadvertent RhIg administration [6], which is consistent with our observations. Serum RhIg concentrations reportedly peak two to seven days after intramuscular injection, with an elimination half-life of approximately 18-31 days (product dependent) [9,11,14,15]. Neonatal RBCs have a shorter lifespan than adult erythrocytes: approximately 55-80 days in healthy term infants compared to 120 days in adults [16,17]. The timing of DAT negativity in our case broadly coincided with the natural lifespan of neonatal RBCs, suggesting that antibody-coated RBCs were not rapidly destroyed but were gradually cleared from circulation in accordance with their natural senescence.

Recommendations for clinical management

Inadvertent RhIg administration in Rh(D)-positive neonates is unlikely to result in severe hemolytic complications. However, because the potential for exacerbated physiological jaundice or progressive anemia cannot be entirely ruled out, close monitoring is required. In line with previous reports, we consider inpatient monitoring for approximately one week post-exposure, followed by an outpatient evaluation at approximately one month of age, to be a reasonable management strategy.

## Conclusions

We encountered a case in which RhIg intended for an Rh(D)-negative mother was inadvertently administered to an Rh(D)-positive neonate. Although the patient exhibited prolonged DAT positivity, no serious adverse events such as hemolytic anemia or severe hyperbilirubinemia were observed. Our findings, together with prior literature, suggest that such inadvertent administrations can be managed safely through careful observation and regular outpatient follow-up.
